# Clinical benefit of readministration of gefitinib for initial gefitinib-responders with non-small cell lung cancer

**DOI:** 10.1186/1471-2407-7-51

**Published:** 2007-03-20

**Authors:** Hiroshi Yokouchi, Koichi Yamazaki, Ichiro Kinoshita, Jun Konishi, Hajime Asahina, Noriaki Sukoh, Masao Harada, Kenji Akie, Shigeaki Ogura, Takashi Ishida, Mitsuru Munakata, Hirotoshi Dosaka-Akita, Hiroshi Isobe, Masaharu Nishimura

**Affiliations:** 1 First Department of Medicine, Hokkaido University School of Medicine, Sapporo, Japan; 2 Department of Medical Oncology, Hokkaido University Graduate School of Medicine, Sapporo, Japan; 3 Department of Respiratory Medicine, National Hospital Organization Hokkaido Cancer Center, Sapporo, Japan; 4 Department of Respiratory Disease, Sapporo City General Hospital, Sapporo, Japan; 5 Department of Pulmonary Medicine, Fukushima Medical University, Fukushima, Japan; 6 Department of Medical Oncology, KKR Sapporo Medical Center, Sapporo, Japan; 7 Hokkaido Lung Cancer Clinical Research Group, Sapporo, Japan

## Abstract

**Background:**

Gefitinib, an oral agent of epidermal growth factor receptor tyrosine kinase inhibitor, has a certain efficacy against non-small cell lung cancer (NSCLC). Several predictive factors of gefitinib sensitivity have been well described. However, few studies have investigated the clinical features of gefitinib-responders. In the present study, we analyzed the response and disease progression of primary and metastatic lesions to gefitinib in responders and the results of gefitinib readministration following temporary cessation of gefitinib upon progression of initial gefitinib treatment and other treatments.

**Method:**

We retrospectively evaluated the clinical courses of 27 NSCLC patients who received gefitinib and achieved either a complete or partial response.

**Results:**

The best-response rate and disease-control rate against the initial chemotherapy for the gefitinib-responders were 27.3% and 77.3%, respectively. Favorable efficacy was observed in the primary lesion and metastases to the lung, liver and brain, while there was no obvious effect on bone metastasis. The primary lesion and intrapulmonary metastasis were the sites of major recurrence. Median progression-free survival was 13.8 months, median duration of gefitinib treatment was 17.0 months and median overall survival was 29.2 months. Some of the patients who experienced disease progression after responding to gefitinib were again sensitive to readministration of gefitinib following temporary cessation of gefitinib and other treatments.

**Conclusion:**

Patients may still be expected to have prolonged survival if they once responded to gefitinib and then underwent various subsequent treatments followed by readministration of gefitinib. These findings might provide valuable information for the management of gefitinib-responders.

## Background

Although chemotherapy improves survival in advanced NSCLC patients, it appears to have reached a therapeutic plateau and novel approaches are urgently required. Under these circumstances, inhibition of the epidermal growth factor receptor (EGFR) tyrosine kinase has emerged as a therapeutic option in patients with NSCLC. Gefitinib, an oral EGFR tyrosine kinase inhibitor (EGFR-TKI), is a leading agent in this class of novel therapeutic agents. It is now clear that there are limited subgroups of patients who derive particular benefit from this treatment. Two major phase II trials [1,2], large expanded access programs across the world [3-6] and other studies [7,8] have demonstrated a higher objective-response rate and prolonged survival in women, never-smokers, patients with adenocarcinoma, and East-Asian patients. Moreover, a prospective trial using gefitinib as a first-line therapy for advanced lung adenocarcinoma patients with never smoking status was conducted in Korea, with excellent efficacy confirmed [9].

A molecular approach linked to gefitinib sensitivity has also been attempted. Mutations [10,11] and amplification [12] of the EGFR gene, and other molecules such as phosphorylated Akt [13] and ErbB-3 expression [14] have been well described as markers of a better outcome in patients treated with gefitinib. Furthermore, correlation between some of these predictors and clinical benefit has been retrospectively confirmed in many reports [15-22]. However, few reports have examined the clinical courses of gefitinib-responders.

In the present study, we investigated the clinical courses of gefitinib responders, by analyzing the response and disease progression of primary and metastatic lesions by gefitinib in responders and the results of gefitinib readministration (resumption of, or rechallenge with, gefitinib) following temporary cessation of gefitinib upon progression of initial gefitinib treatment and other treatments. Our results provide additional information regarding gefitinib use for advanced NSCLC patients.

## Methods

### Patients and treatment

From August 2002 to March 2004, a total of 122 patients with histologically or cytologically confirmed advanced NSCLC received 250 mg/day gefitinib orally at our institutes (Hokkaido University Hospital, National Hospital Organization Hokkaido Cancer Center, Sapporo City General Hospital and Fukushima Medical University Hospital, Japan). We reviewed their medical records and imaging findings. The histopathological classification was based on WHO criteria [23]. Treatment was primarily continued until disease progression (PD), death, intolerable side effects determined by physicians or withdrawal of consent. No other systemic chemotherapy was performed during gefitinib treatment in any of the patients, although some received radiotherapy for metastasis or pleurodesis after temporary cessation of gefitinib, followed by resumption of gefitinib.

The clinical features of the 27 (22.1%) patients who were defined as having a complete (CR) or partial response (PR) to gefitinib were retrospectively analyzed. Objective tumor response was determined in accordance with the Response Evaluation Criteria in Solid Tumors Group (RECIST) guidelines [24]. Disease control was categorized as CR, PR, or stable disease (SD). CR and PR required a sustained response for 4 weeks or longer, while this was 8 weeks or longer for SD. Most patients who started gefitinib were admitted to hospital and underwent weekly chest X-rays for the initial 4 weeks, and then underwent monthly chest X-rays and computed tomography (CT) including target lesions with or without brain magnetic resonance imaging (MRI) or bone scintigraphy every three months at the outpatient clinic. Objective tumor response was confirmed by CT, MRI, and bone scintigraphy. Progression-free survival (PFS) and overall survival (OS) were calculated from the date of initiation of gefitinib. Patients who were not deceased were censored at the date of last contact with our institutions. The duration of gefitinib treatment was calculated from the date of initiation of gefitinib to the date of withdrawal of gefitinib. In the present study, readministration of gefitinib was divided into resumption and rechallenge of gefitinib. "Resumption of" or "resuming" gefitinib indicates the restarting of gefitinib administration within 4 weeks after cessation of gefitinib and other treatments such as radiotherapy for metastasis or pleurodesis, but not chemotherapy. "Rechallenge" with gefitinib indicates the restarting of gefitinib after cessation of gefitinib and cessation of several cycles of other chemotherapy.

### Statistical analysis

PFS and OS probability estimates were based on the Kaplan-Meier method. Confidence intervals were calculated at the 95% level (95% CI). Dr. SPSS software (SPSS, Inc., Chicago, IL) was used for the analyses.

## Results

### Differential response and disease progression of primary and metastatic lesions by gefitinib

Of the entire cohort of 122 patients who were treated with gefitinib, 27 (22.1%) were defined as PR. No patient achieved CR. Female patients, patients with never-smoked status and patients with adenocarcinoma showed a higher response as described previously [7].

Table 1 shows the response of primary and metastatic lesions to gefitinib in the responders. The primary tumor shrunk by 46.5 ± 19.4% (mean ± SD) in the 21 assessable gefitinib-responders. At the start of gefitinib administration, 12 patients (44.4%) had intrapulmonary metastases, all of which regressed by gefitinib. The intrapulmonary metastases in 9 patients were assessed as target lesions and the percentage of shrinkage of these target lesions was 73.8 ± 30.6%. The response of liver and brain metastases, and pleural effusion, to gefitinib was also favorable. Bone metastasis was initially detected in 8 patients (29.6%), and remained unchanged after gefitinib treatment, as confirmed by bone scintigraphy.

The sites of initial disease progression after response to gefitinib among the 27 responders are summarized in Table 2. The initial recurrent sites were seen in the primary lesion in 8 (29.6%) patients, intrapulmonary lesion in 8 (29.6%), bone in 5 (18.5%), central nervous system (CNS) in 3 (11.1%), and pleural effusion in 6 (22.2%).

### Gefitinib readministration following temporary cessation of gefitinib upon progression of initial gefitinib treatment and other treatments

Table 3 shows the clinical courses of initial gefitinib-responders upon progression of initial gefitinib treatment. At the time of writing, 3 responders are maintaining PR and remain on gefitinib. Two patients have ceased gefitinib during PR at their request, and the other 22 patients have developed PD. Of these 22, 4 continued to receive gefitinib at their request and regardless of PD and the availability of other treatments.

Another 4 of the 22 patients with PD underwent various treatments, except chemotherapy, for new or progressed lesions and then resumed gefitinib (Table 4). Patient 1 developed a new bone metastasis and increment of malignant pleural effusion regardless of regression of intrapulmonary tumors. This patient received irradiation for the bone metastasis, pleurodesis, and then resumed gefitinib. Patient 2 developed a brain metastasis and thus underwent whole brain irradiation, after which gefitinib was resumed because intrathoracic disease remained well controlled. Patient 3 developed a new bone metastasis regardless of good control of intrathoracic disease. The patient underwent irradiation for the bone metastasis and then resumed gefitinib. Patient 4 who had well-controlled intrapulmonary tumors, underwent irradiation for brain and bone metastases, pleurodesis, and then gefitinib again. Judging from the dates of resumption, all 4 patients were confirmed as SD. The range of time to re-progression was from 2.9 months to more than 19.1 months.

Nine of the 22 patients underwent systemic chemotherapy when reaching PD. Of these 9 patients, 6 chose to receive gefitinib again after chemotherapy (Table 5). Judging from the point of rechallenge of gefitinib, one patient achieved PR, three patients SD, one patient PD and one patient was not evaluative (NE). The range of time to re-progression from gefitinib rechallenge was 0.6 months to 7.8 months.

**Figure 1:**
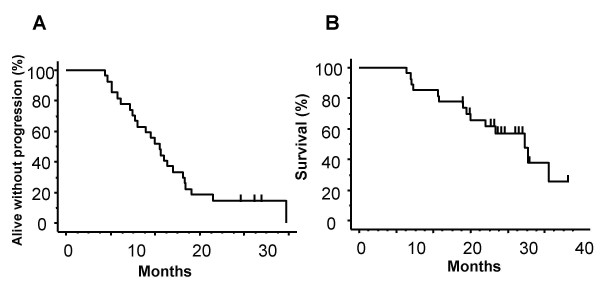
**Kaplan-Meier estimates of progression-free survival (PFS, A) and overall survival (OS, B) for gefitinib-responders**. Median PFS was 13.8 months (95% CI = 11.4 to 16.2 months) and median OS was 29.2 months (95% CI = 22.1 to 36.4 months). Bars indicate censored cases.

In the present cohort of 122 patients, the median follow up of the 27 initial gefitinib-responders was 23.7 months, the median PFS,13.8 months (95% CI = 11.4 to 16.2 months); and the median OS, 29.2 months (95% CI = 22.1 to 36.4 months) (Figure 1). The estimated 1-year survival rate was 85.2%. Because many patients continued to take gefitinib even after disease progression, the median duration of gefitinib treatment was 17.0 months (95% CI = 13.2 to 20.8 months).

## Discussion

In the present retrospective study, we analyzed the clinical courses of gefitinib-responders during and after failure of the treatment by analyzing the response and disease progression of primary and metastatic lesions by gefitinib in responders, and the results of gefitinib readministration following temporary cessation of gefitinib upon progression of initial gefitinib treatment and other treatments.

The median PFS and median duration of gefitinib treatment were 13.8 months and 17.0 months, respectively, revealing an approximately 3.2 month difference between PFS and the time to cessation of gefitinib. In some patients, other treatments against pleural effusion, or against bone or brain metastasis, were successful, while intrapulmonary or intrathoracic diseases were stable. Physicians and patients decided together whether to continue, resume or rechallenge with gefitinib.

One of the most striking results in the present study was that PR or SD was achieved upon gefitinib rechallenge following systemic chemotherapy in 4 of 6 patients who responded to the first administration of gefitinib. There has been only one similar case reported [25]. This phenomenon is difficult to explain. One possibility is that the passage of time or cytotoxic chemotherapy may have reduced the number of clones containing modified genes and proteins that confer resistance to gefitinib. The mechanism of this phenomenon may involve a second point-mutation, resulting in a threonine-to-methionine amino acid change at position 790 of EGFR (T790M) [26,27], mutation in the KRAS protein [28], or upregulation of epithelial membrane protein-1 [29]. It would be of great benefit to perform comparative molecular analyses of tissue specimens before and after gefitinib treatment in responders.

In the present study, the OS was 29.2 months, which was much longer than that in three previous retrospective reports [19,30,31] focusing on patients who had responded to gefitinib (16 to 20.3 months). Differences in gender, never-smoked status, presence of adenocarcinoma, and ethnicity may be responsible in part for the altered outcome between the responders in the present study and those in the three previous studies. Moreover, the strategy of resumption or rechallenge of gefitinib for gefitinib-responders may also have played a role in the longer survival observed in the present study.

Hotta et al. [32] noted the importance of achieving SD with gefitinib, based on their finding that the OS in patients who obtained SD was significantly longer than that in patients with PD. It is of interest whether resumption of, or rechallenge with, gefitinib after treatment of a recurrence site contributes to the patients' survival by achieving PR or maintaining SD for a long time. With regard to rechallenge, we are performing a prospective phase II trial of gefitinib rechallenge to gefitinib-responders following chemotherapy.

Our analysis of the initial sites of disease recurrence in patients who initially responded to gefitinib revealed a relatively higher prevalence of intrapulmonary and primary lesion metastasis at the initial disease recurrence site than at other organs. Furthermore, although a previous report [30] noted that CNS metastasis was a frequently observed disease recurrence site (33%) in gefitinib-responders, only 3 patients (11.1%) developed CNS metastasis in the present study. There were more patients who had pleural effusion or bone metastasis at the initiation of gefitinib in our cohort.

## Conclusion

Patients may still be reasonably expected to have prolonged survival if they once responded to gefitinib and then underwent various subsequent treatments followed by failure of a second round of gefitinib. These findings provide valuable information for the management of gefitinib-responders. Further research and clinical trials based on the present findings will be needed to develop more effective treatment strategies and to improve the clinical practice of appropriate gefitinib administration in advanced NSCLC patients.

## Competing interests

The author(s) declare that they have no competing interests.

## Authors' contributions

HY reviewed the medical records and imaging findings, drafted the manuscript and coordinated the submission. KY designed the analysis and critically revised the manuscript. IK, HDA, and MN participated in reviewing the manuscript. JK, AH, NS, MH, KA, SO, TI, MM, and HI followed the patients and participated in the acquisition of data. All authors read and approved the final manuscript.

## Pre-publication history

The pre-publication history for this paper can be accessed here:



## Figures and Tables

**Table 1:** The response of primary and metastatic lesions to gefitinib in responders

Site	No. of patients	No. improved	No. of patients with target lesion	Average tumor shrinkage of target lesion (%, mean ± SD)
Primary lesion	27	27	21	46.5 ± 19.4
Metastatic lesion				
Intrapulmonary	12	12	9	73.8 ± 30.6
Liver	4	4	3	47.3 ± 21.6
CNS	4	4	2	65.0 ± 21.2
Bone	8	0		
Pleural effusion	6	5		
Others	2	2		

CNS, central nervous system

**Table 2:** The sites of initial disease progression after response to gefitinib

Site	No. of patients	%
Primary lesion	8	29.6
Metastatic lesion		
Intrapulmonary	8	29.6
Pleural effusion	6	22.2
Bone	5	18.5
CNS	3	11.1
Others	0	0

CNS, central nervous system

**Table 3:** Clinical course of gefitinib-responders

Clinical course	No. of patient
Maintain PR and still continue	3
Cessation by patient's will during PR	2
Disease progression (PD)	22
Insistence to continue	4
Resumption	4
Rechallenge	6
Cessation of gefitinib by PD and no therapy	8

**Table 4:** Clinical course of the gefitinib-responders who achieved PD, underwent various treatments except for chemotherapy, and resumed gefitinib

No.	Treatment after gefitinib failure	Response after gefitinib resumption	Time to re-progression (month)
1	RT to bone mets, Pleurodesis,	SD	2.9
2	RT to brain mets,	SD	8.9
3	RT to bone mets,	SD	19.1+
4	RT to bone and brain mets, Pleurodesis,	SD	6.5

"Resumption" of gefitinib indicates restarting gefitinib within 4 weeks after temporary cessation of gefitinib. RT, radiotherapy; mets, metastasis; Time to re-progression, time to progression from the date of resuming gefitinib; +, still continuing the response at the last date of follow-up

**Table 5:** Clinical course of the gefitinib-responders who achieved PD, underwent chemotherapy, and rechallenged with gefitinib

No.	Chemotherapy regimen after failure of gefitinib	Best response of the chemotherapy	Response of gefitinib rechallenge	Time to re-progression (month)
5	GEM+VNR	PR	PR	6.2
6	GEM+VNR	PD	SD	7.8
7	GEM+DTX	SD	PD	0.6
8	CBDCA+CPT-11 → AMR → S-1	SD	SD	1.9+
9	DTX	SD	SD	4.3+
10	DTX	SD	NE	0.9+

"Rechallenge" of gefitinib indicates restarting gefitinib after cessation of gefitinib followed by other chemotherapy. GEM, gemcitabine; VNR, vinorelbine; DTX, docetaxel; CBDCA, carboplatin; CPT-11, irinotecan; AMR, amrubicin; Time to re-progression, time to progression from the date of rechallenging gefitinib; +, still continuing the response at the last date of follow-up
